# Numerical investigation of nanofluid flow using CFD and fuzzy-based particle swarm optimization

**DOI:** 10.1038/s41598-021-00279-6

**Published:** 2021-10-25

**Authors:** Rahmad Syah, Marischa Elveny, Mahyuddin K. M. Nasution, Vadim V. Ponkratov, Mariya Yurievna Kuznetsova, Andrey Leonidovich Poltarykhin, Meisam Babanezhad

**Affiliations:** 1 Data Science & Computational Intelligence Research Group, Universitas Medan Area, Medan, Indonesia; 2 Data Science & Computational Intelligence Research Group, Universitas Sumatera Utara, Medan, Indonesia; 3 Financial University Under the Government of the Russian Federation, Moscow, Russian Federation; 4 Sechenov First Moscow State Medical University, Moscow, Russia; 5 Plekhanov Russian University of Economics, 117997 Moscow, Russia; 6 Department of Energy, Faculty of Mechanical Engineering, South Tehran Branch, Islamic Azad University, Tehran, Iran; 7 Department of Artificial Intelligence, Shunderman Industrial Strategy Co., Tehran, Iran

**Keywords:** Engineering, Chemical engineering, Mechanical engineering

## Abstract

This paper is focused on the application and performance of artificial intelligence in the numerical modeling of nanofluid flows. Suspension of metallic nanoparticles in the fluids has shown potential in heat transfer enhancement of the based fluids. There are many numerical studies for the investigation of thermal and hydrodynamic characteristics of nanofluids. However, the optimization of the computational fluid dynamics (CFD) modeling by an artificial intelligence (AI) algorithm is not considered in any study. The CFD is a powerful technique from an accuracy point of view. However, it could be time and cost-consuming, especially in large-scale and complicated problems. It is expected that the machine learning technique of the AI algorithms could improve such CFD drawbacks by patterning the CFD data. Once the AI finds the CFD pattern intelligently, there is no need for CFD calculations. The particle swarm optimization-based fuzzy inference system (PSOFIS) is considered in this study to predict the velocity profile of Al_2_O_3_/water turbulent flow in a heated pipe. One of the challenging problems in CFD modeling is the lost data for a specific boundary condition. For example, the CFD data are available for wall heat fluxes of 75, 85, 105, and 125 w/m^2^, but there is no data for the wall heat flux of 95 w/m^2^. So, the PSOFIS learns the available CFD data, and it predicts the velocity profile for where the data is not available (i.e., wall heat flux of 95 w/m^2^). The intelligence of PSOFIS is checked by the coefficient of determination (R^2^ pattern) for different values of accept ratio (AR) and inertia weight damping ratio (IWDR). The best intelligence is obtained for the AR and IWDR of 0.7 and 0.99, respectively. At this condition, the velocity profile predicted by both CFD and PSOFIS is compatible. As the performance of the PSOFIS, for learning time of 268 s, the prediction of the CFD data lost was negligible (~ 1 s). In contrast, the CFD calculation takes around 600 s for each simulation.

## Introduction

Based on the studies on thermal features, it is indicated that a weak thermal conductivity is exhibited by all today's liquid coolants utilized as heat transfer fluids compared to solid metals. Limited enhancement can be obtained by supplementary attempts to increment the heat transfer coefficient by incrementing area, agitation, or solid dispersion by the fluid’s low thermal conductivity. Hence, it is rational that attempts are based on increasing the cooling fluid’s thermal conduction performance. There have been former attempts on incrementing the base fluids’ thermal conductivity by suspending solid particles in macro sizes in fluids due to the solid’s thermal conductivity typically 2–3 times higher than the liquids. Nevertheless, the addition of micrometer size particles results in various problems causing sedimentation, clogging, erosion, and pressure drop in channels, conduits, or pipes. As the new multi-element fluids, nanofluids comprise solid particles with their distinctive sizes (< 100 nm). Their dispersion in pure fluids alters the viscosity and thermal conductivity of the solid nanoparticles. The benefits of utilizing the nanoparticles dispersed within a base fluid in different thermal systems were provided^1–11^. In one study, the flow characteristics and convective heat transfer for a Cu–water nanofluid flowing within a constant heat flux straight tube at turbulent and laminar flow circumstances were experimentally assessed^12^. The findings indicated that the suspended nanoparticles considerably improved the conventional base fluid’s heat transfer behavior, and there was good consistency between their friction factor and the water. Moreover, they proposed novel convective heat transfer correlations to predict the nanofluid’s heat transfer coefficients for both turbulent and laminar flow circumstances. The effects of SiO_2_ nanoparticles on heat transfer in a cavity were numerically analyzed^13^. In this work, an experimental setup was used for determining the nanofluid’s thermal conductivity. Another study tested the convective heat transfer coefficients of CuO–water and Al_2_O_3_–water nanofluids in laminar flow^11^. Results showed that increasing the concentration of the solution increased the heat transfer coefficient. Quantitative research found that the Ethylene Glycol–Water mixes provide different effects on heat transfer for turbulent flows of Al_2_O_3_, SiO_2_, and CuO nanoparticles in the presence of varying volume concentrations running in a tube under constant heat flux settings^14^. The increase in nanoparticle volume concentration induces an increase in heat transfer coefficient.

The numerical approaches are usually used to estimate the empirical variables and avoid the experiments' implementation cost. Numerous research papers have already studied the computational fluid dynamics (CFD) modeling of nanofluids^15–21^. Almost all investigations focused on the CFD model accuracy for the prediction of heat and fluid flow variables. The CFD must discretize and solve many partial differential equations describing the governing equations for finite volume cells of the fluid flow domain. Although the CFD is known as a robust method for predicting heat transfer and fluid dynamics parameters, this method requires a lot of computational time and expenses for complex CFD problems (i.e., large geometries, turbulent flows, etc.). The use of CFD techniques in artificial intelligence has gained attention in the last few years. An adaptive network-based fuzzy inference system (ANFIS) was also credited with the contribution of artificial intelligence to CFD in a few types of research^22–28^. The results released the efficiency of the ANFIS for the accurate predictions of the CFD results. However, a deep gap is seen for investigating any other algorithms and their tuning parameters for the best intelligence. In addition, the accuracy of the AI algorithm for the prediction of the lost CFD data is examined. AI algorithms can train the CFD results.

The performance of the CFD models can be predicted when the boundary conditions of the CFD examples are adjusted. This work is designed to address the aforementioned research gap, in part, using a particle swarm optimization (PSO) algorithm paired with a CFD simulation. The prediction of the velocity profile of Al_2_O_3_/water turbulent flow in a heated pipe is the main objective of this study.

## Methodology

### CFD approach

The flow research used a nanofluid solution in a straight tube. The tube length is one meter, and its diameter is 0.01 m. The tube is under a constant wall heat flux. The fluid is introduced into the tube with consistent axial temperature and velocity. This is a highly nonlinear fluid dynamic problem by considering turbulence effect and temperature-dependent nanofluid thermophysical properties. This problem was solved using the computational fluid dynamics code of the commercial ANSYS-Fluent 14.5 software. The continuity of mass, momentum, and energy are considered for many finite volume cells. These governing equations are also coupled with the turbulence model equations. As a result, many nonlinear partial differential equations were made. The leading equation systems (1)–(3) must be discretized and solved using the control volume method. By the control-volume method, the governing equations are converted to algebraic equations that can be numerically solved. The heading equations for the fluid flow include^29,30^:

Continuity equation:

1 $$\nabla \cdot \left({\rho }_{f}V\right)=0$$

Momentum equation:

2 $$\nabla \cdot \left({\rho }_{f}VV\right)=-{\nabla }_{p}+\nabla \cdot \tau$$

Energy equation:

3 $$\nabla \cdot \left({\rho }_{f}V{C}_{p\cdot f}T\right)=\nabla \cdot \left({k}_{f}\nabla T-{C}_{p\cdot f{\rho }_{f}}\overline{VT}\right)$$

The k–ε turbulence model is based on previously published research and is used to determine the turbulent eddy viscosity, energy dissipation rate $(\varepsilon$), and kinetic energy (k)^16,18,31^.

For modeling such a case, the ANSYS-Fluent 14.5 CFD software is used. This CFD package software works based on the finite volume method (FVM). For discretization, the second-order-upwind scheme is used for the continuity and k–ε turbulence equations. The SIMPLE algorithm is adopted as the pressure and velocity coupling scheme. The scaled residuals for velocity components and energy are equal to 10^–9^.

### PSO algorithm

PSO is a method based on a stochastic optimization population. PSO optimization procedure is initiated by a population of particles or solutions selected randomly in the search space, consequently looking for optima by iterative updating of generations. Each particle is updated based on two higher and outstanding compared to the gbest fitness; updating the gbest fitness factors is essential. In the second state, where the particle fitness is higher and superior to the pbest fitness, it is essential to update the related parameters of pbest fitness. Ultimately, based on the second phase again the further particles should be assessed.

### Fuzzy Inference System (FIS)

Fuzzy inference is widely used in computers, particularly in fuzzy reasoning, fuzzy set theory, and if–then logic. While it was widely adopted in many different areas, the technique was hard to apply in domains that required interaction design or application software. FIS can carry out three separate kinds of fuzzy reasoning, using the if–then rules used by Takagi and Sugeno^32^. To get velocity as output, the three input variables of x coordinate, y coordinate, and wall heat flux (Q_wall_) are used. Incoming signals from the last stage are multiplied according to the AND rule. For case, the i-th rule function is

4 $${w}_{i}={\mu }_{Ai}\left(X\right) {\mu }_{Bi}\left(Y\right){\mu }_{Ci}(Qwall)$$

The w_i_ variable represents the signal output from the node while ${\mu }_{Ai}$, ${\mu }_{Bi}$ and ${\mu }_{Ci}$ indicate the input signals, containing X, Y, and, the Q_wall_ value.

For the next stage, each rule’s firing strength is estimated relative to the total weight of all rules:

5 $$\overline{{w }_{i}}=\frac{{w}_{i}}{\sum \left({w}_{i}\right)}$$

Node function can be written as:

6 $$\overline{{w }_{i}}{f}_{i}=\overline{{w }_{i}}({p}_{i}X+{q}_{i}Y+{r}_{i}Qwall+{s}_{i})$$

Consequent parameters of the if–then rules, often called p_i_, q_i_, r_i_, and s_i_, respectively. To compute the estimate result, the signals of the last stage are used. In this instance, the MFs and consequent parameters are both updated using a hybrid learning method that combines gradient descent and LSE techniques^33,34^.

## Results

The suspension of metallic nanoparticles can enhance the heat transfer of water. Although many studies have investigated the nanofluid fluid flow numerically, there are no investigations to optimize the CFD modeling by the artificial intelligence algorithm. This study aims to include the finite volume method (FVM) results from computational fluid dynamics (CFD) into the learning process of a machine learning system. Models of computational fluid dynamics use partial differential equations to describe mass, momentum, and energy governing equations. When the equations are discretized and arranged within specific boundary conditions, the FVM can solve all of the equations. Solving the governing equations, the independent variables such as velocity, pressure, and temperature are achieved for each node in the domain. The FVM solutions are learned by one of the artificial intelligence (AI) algorithms. Once the best intelligence of the algorithm is obtained, it is no longer necessary to solve the governing equations. The machine learning approach of AI algorithms could save much computational time and cost, especially in large-scale problems. An artificial algorithm could also substitute the CFD modeling for doing further simulations. For example, for CFD data loss, where the CFD data are unavailable for specific boundary conditions, the AI algorithm could predict the lost data based on the pattern of the existing data. When it refers to artificial intelligence algorithms, the particle swarm optimization-based fuzzy inference system (PSOFIS) is one of the most widely utilized. The turbulent flow of Al_2_O_3_/water nanofluid inside a pipe is considered as a case for simulation. The pipe is conducted under different constant wall heat fluxes (Q_wall_). The velocity profile, corresponding to each wall heat flux, is adopted as an independent variable for prediction by the PSOFIS.

**Figure 1:**
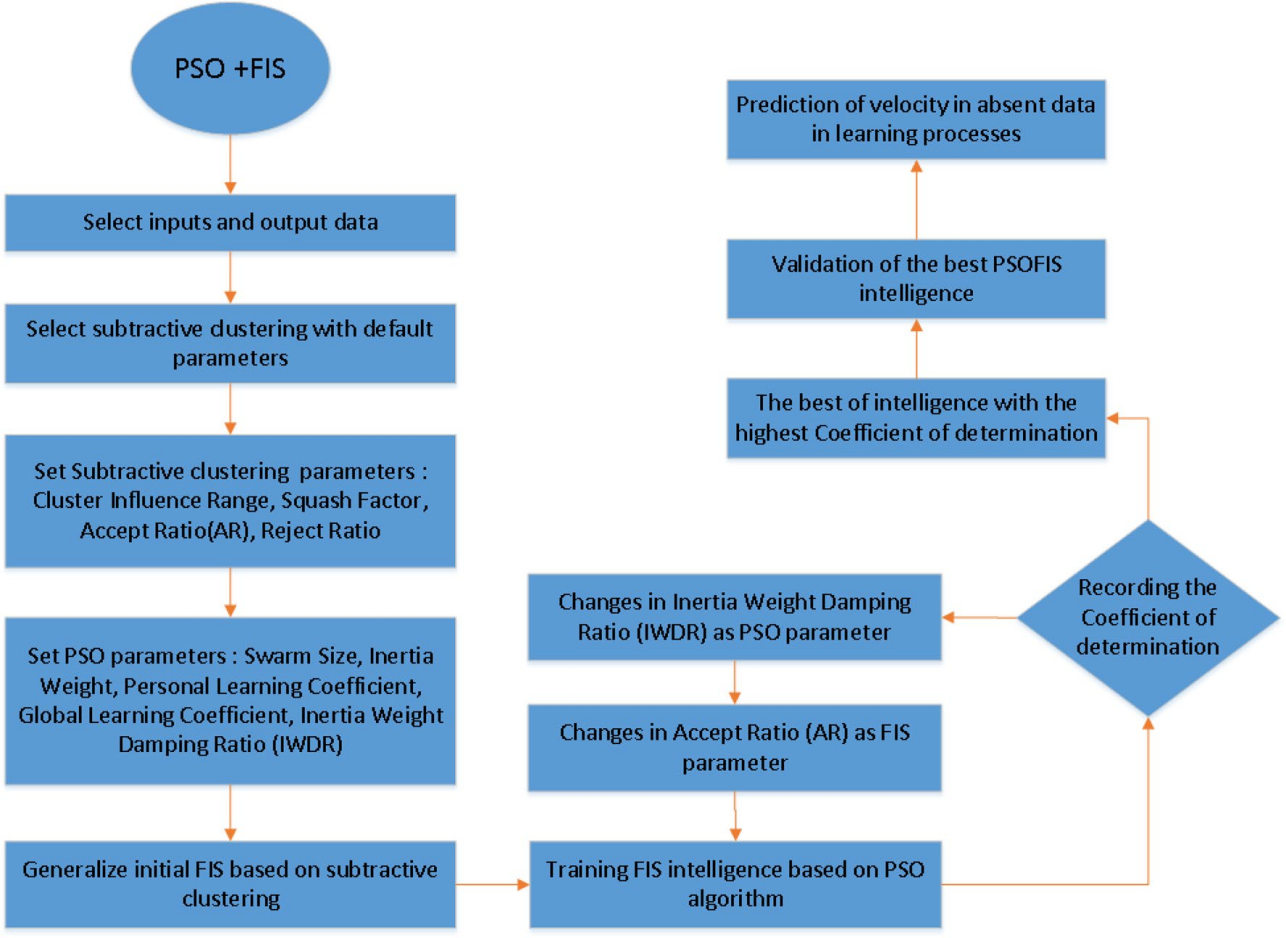
Schematic of PSO + FIS method.

Figure 1 shows different steps for the PSOFIS algorithm setup to predict velocity in the present study. The type of data clustering is subtractive clustering. After adopting the CFD data as inputs (i.e., x, y, and Q_wall_) and output (i.e., nanofluid velocity), the subtractive clustering parameters, including accept ratio, reject ratio, squash factor, and cluster influence range (CIR), are determined. The constant values of 0.15 and 1.25 are considered for reject ratio and squash factor, respectively. The constant values of 0.8, 0.15, and 1.25 are considered for CIR, reject ratio, and squash factor. However, a sensitivity test is done for the effect of accept ratio (AR) on the intelligence of the PSOFIS.

For particle swarm optimization (PSO) parameters, the swarm size is supposed to be constant (i.e., 100), while different values of inertia weight damping ratio (IWDR) are tested for the best intelligence. The fuzzy inference system (FIS) is generalized based on the subtractive clustering parameters. The intelligence of PSOFIS is checked by the coefficient of determination (R2 pattern). The highest coefficient of determination means that the best intelligence has been achieved. The PSOFIS does the CFD data learning for the wall heat fluxes of 75, 85, 105, and 115 w/m^2^. The sensitivity tests are done for different AR values (i.e. 0.3, 0.4, 0.5, 0.6, and 0.7) and IWDR values (i.e. 0.6, 0.7, 0.8, 0.9, and 0.99). The CFD results validate the results of the PSOFIS. After achieving the intelligence, the PSOFIS can predict any CFD data which did not attend in the machine learning (e.g., velocity profile extraction for Q_wall_ = 95 w/m^2^).

**Figure 2:**
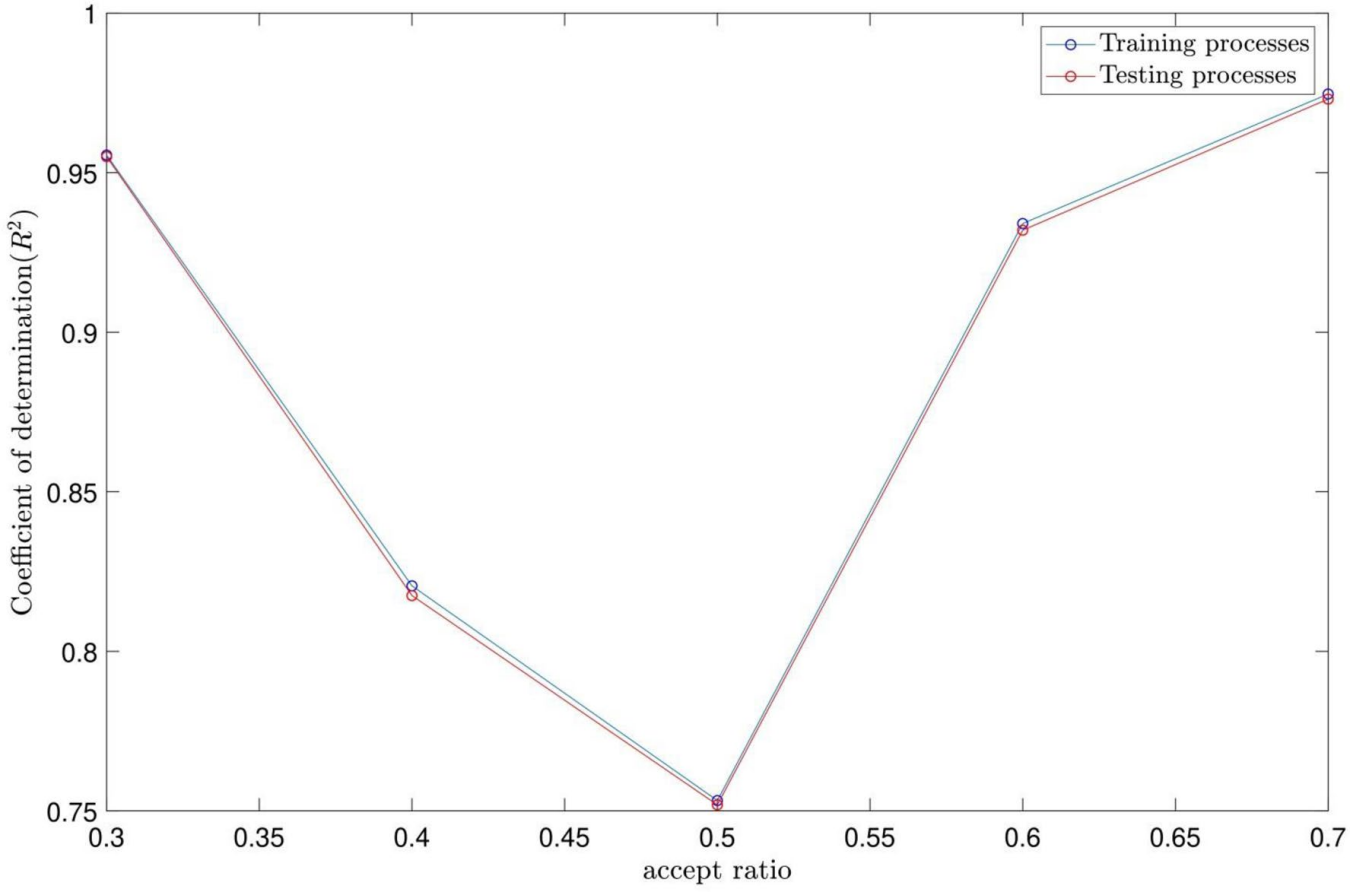
PSO + FIS learning processes with changes in accept ratio as subtractive clustering parameter when number of inputs is 3.

**Figure 3:**
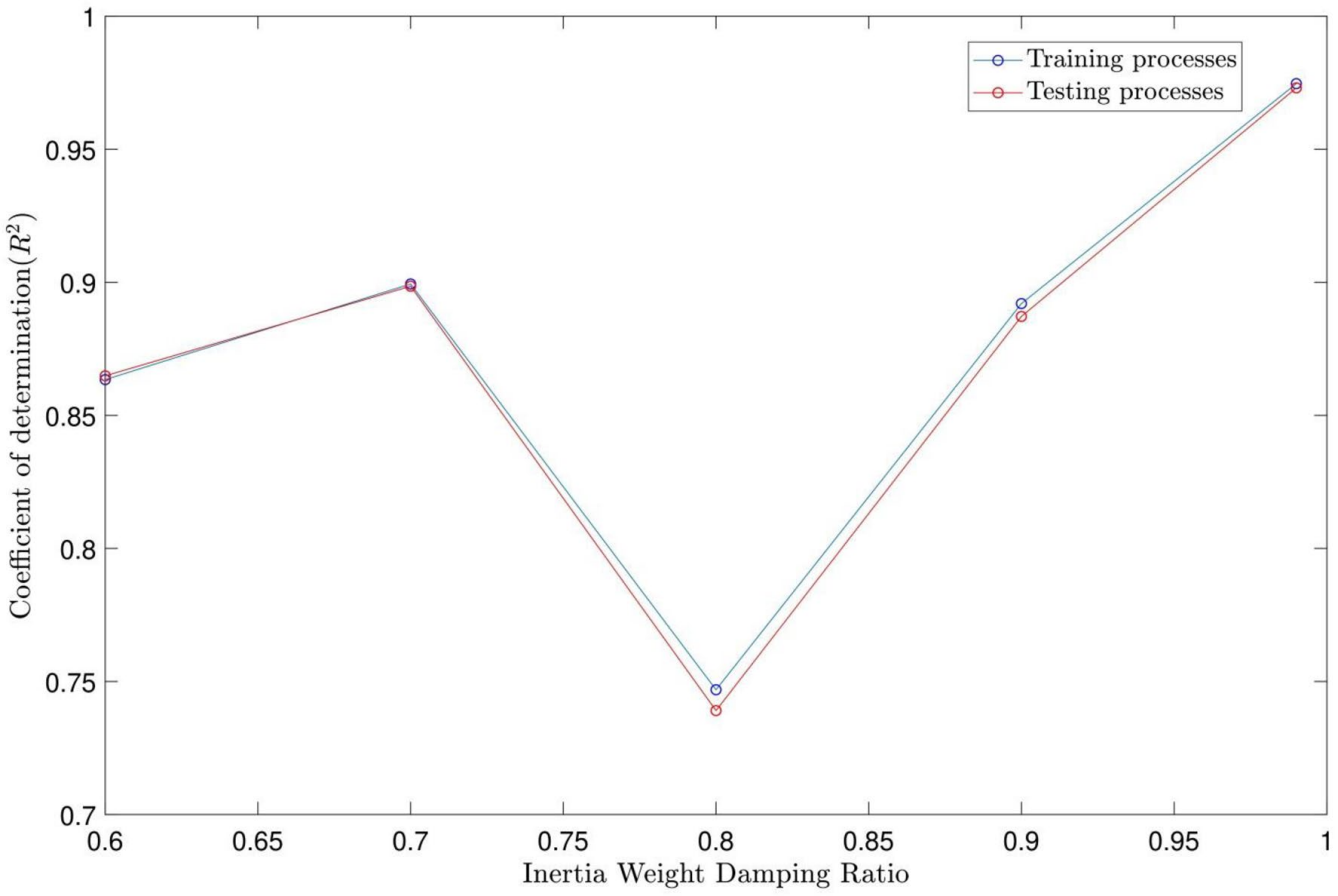
PSO + FIS learning processes with changes in Inertia Weight Damping Ratio as PSO parameter when number of inputs is 3 and accept ratio is 0.7.

**Figure 4:**
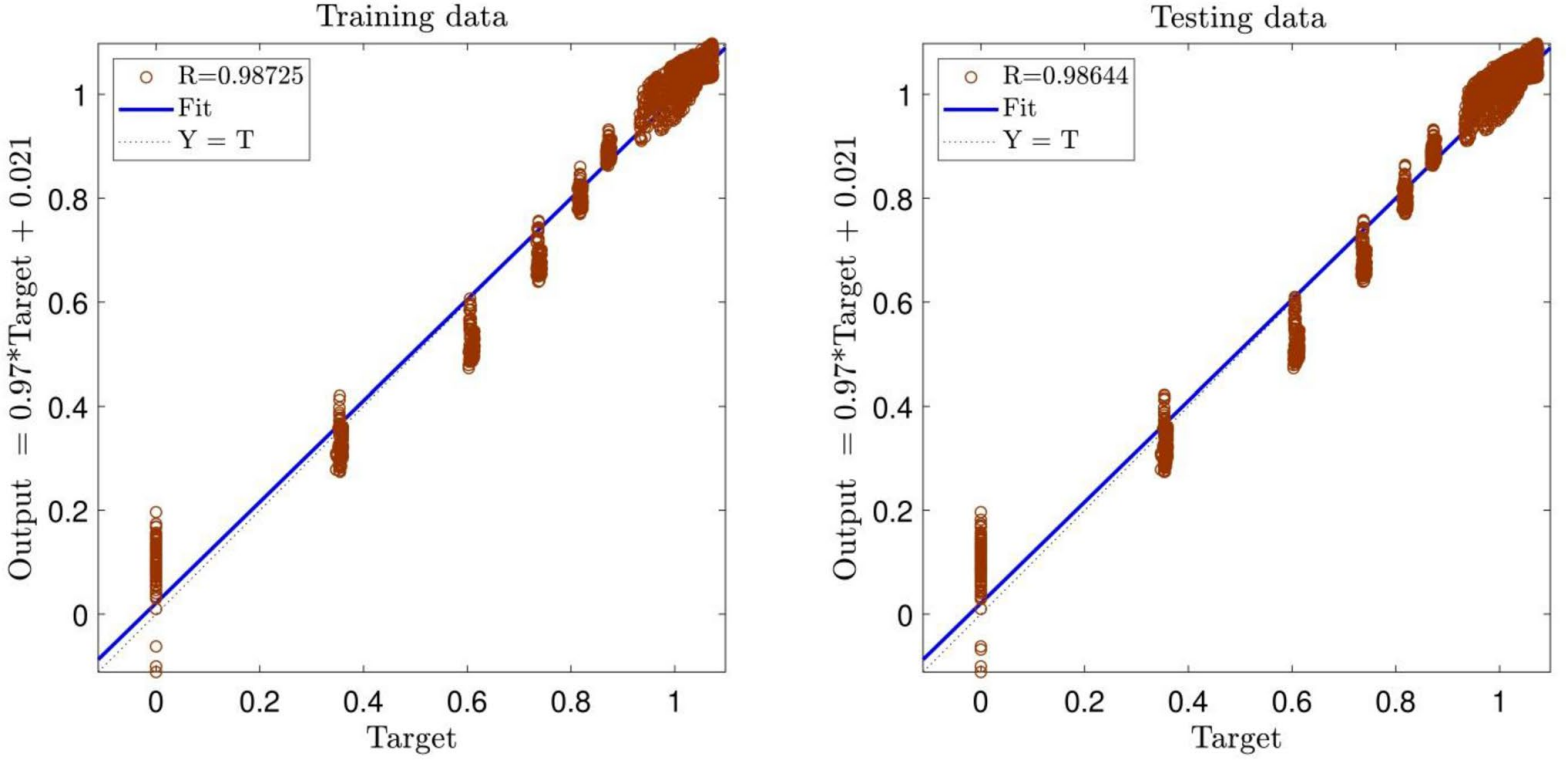
Correlation coefficient in the best PSO + FIS intelligence when number of inputs is 3, accept ratio is 0.7 and inertia weight damping ratio is 0.99.

**Figure 5:**
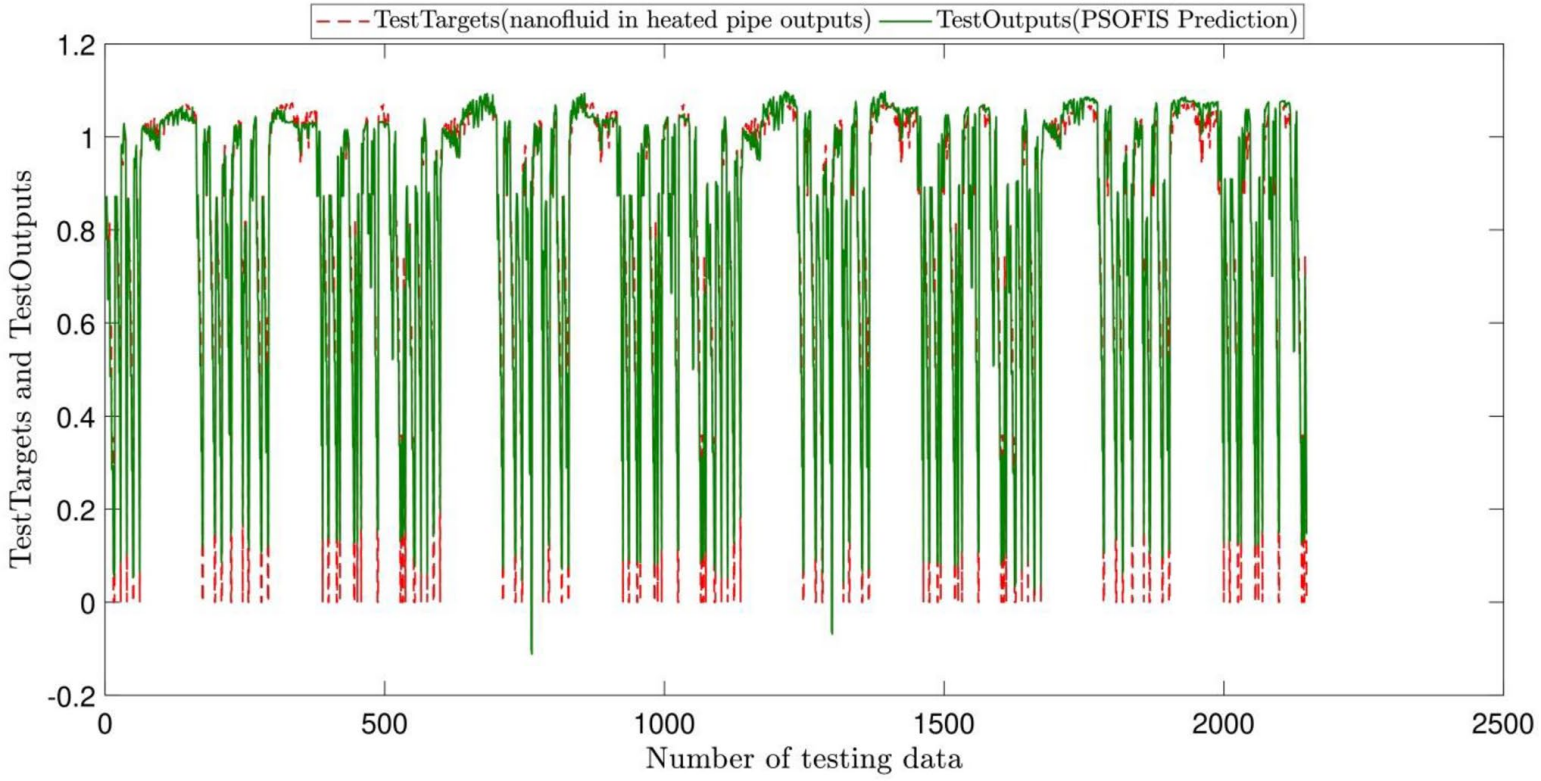
validation of PSO + FIS learning process with comparison between test targets (nanofluid in heated which is CFD output) and PSO + FIS prediction.

According to Figs. 2 and 3, the highest coefficient of determination and, as a result, the best intelligence is obtained for the AR and IWDR of 0.7 and 0.99, respectively. This is confirmed by the regression number of 0.98 for both training and testing processes of the PSOFIS, as shown in Fig. 4. The highest compatibility between the CFD results and the PSOFIS predictions is shown in Fig. 5. The velocity profile extracted by the PSOFIS is the same as that predicted by the CFD.

**Figure 6:**
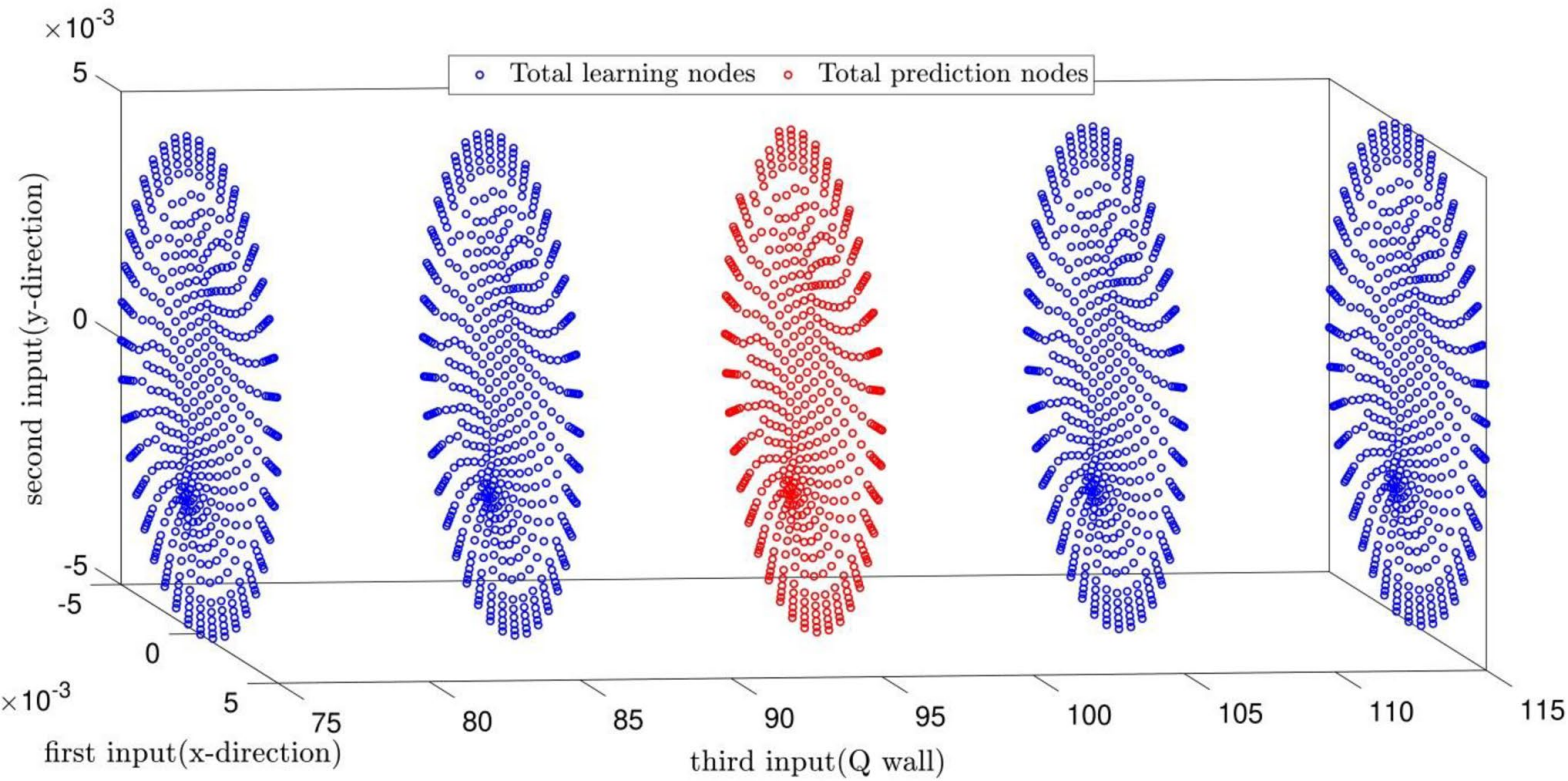
Data which considered in learning processes and prediction data (which not considered in learning processes).

**Figure 7:**
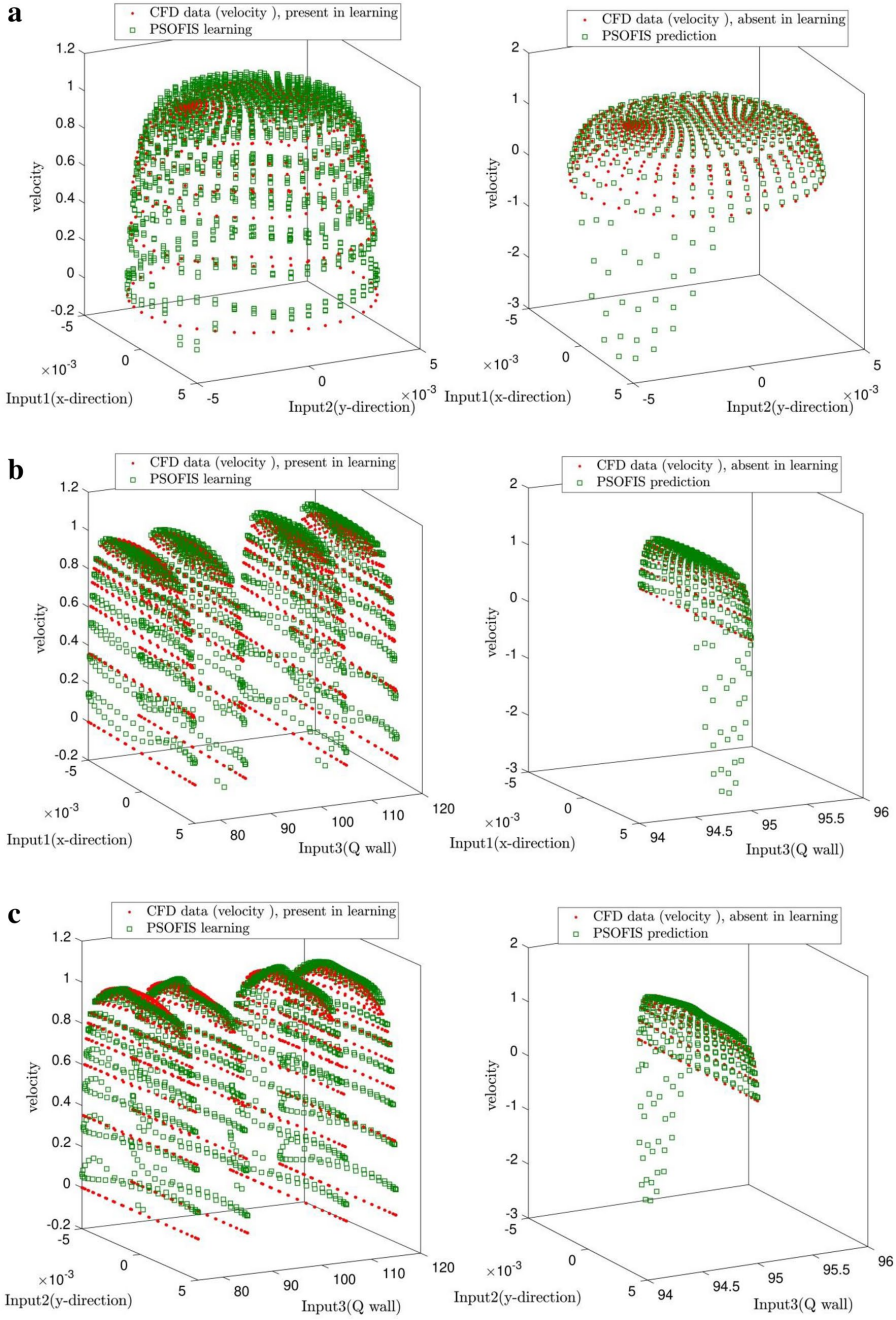
(**a**) Prediction of velocity with absent data when q wall is 95 in right side and learning prediction in left side. (Based on inputs 1 and 2). (**b**) Prediction of velocity with absent data when q wall is 95 in right side and learning prediction in left side. (Based on inputs 1 and 3). (**c**) Prediction of velocity with absent data when q wall is 95 in right side and learning prediction in left side. (Based on inputs 2 and 3).

The CFD data in this study are divided into two types. The first type is the data used in the learning process of the PSOFIS. But the second ones are those just used for prediction. Figure 6 illustrates both types of data. The CFD data for Q_wall_ of 75, 85, 105, and 125 w/m^2^ are used for learning. After achieving the intelligence, the velocity profile is predicted by the PSOFIS for Q_wall_ of 95 w/m^2^, as shown in Fig. 7. The comparison of results between the CFD and the PSOFIS is also illustrated in Fig. 7. The velocity predictions of the CFD and the PSOFIS are in good agreement. Table 1 illustrates the performance of the PSOFIS as the learning (i.e., training and testing) time and the prediction time compared to the CFD time calculation. It shows that after learning the CFD data for a specific time (268 s), the prediction of the data lost could be made in little time (1 s). In contrast, the CFD calculation takes around 600 s for each simulation. So, after mapping the CFD data and achieving the intelligence, the PSOFIS saves computational time. In another comparison, the computer hardware requirements are considered. The PSOFIS calculations could be carried out by typical computer specifications (Intel Core i5 CPU 650 @ 3.20 GHz, 3333 MHz, 2 Cores). In contrast, a workstation computer (Intel Xeon CPU E5-2685 v3 @ 2.60 GHz, 12 Cores) is required for CFD modeling.

**Table 1:** PSOFIS processes time.

Processes	Time(s)
PSOFIS training and testing	268.0254
PSOFIS prediction	1.0145
CFD	600

## Conclusions

This study is aimed to investigate the application and performance of artificial intelligence (AI) algorithms on facilitating CFD calculations of modeling nanofluid flows. The flow simulation was conducted for varying constant wall heat fluxes to see how they influence the turbulent flow of Al_2_O_3_/water within a pipe. The finite volume method (FVM) solved the partial differential equations of mass, momentum, and energy. Fuzzy, artificial intelligence-based particle swarm optimization (PSOFIS) method was also used to apply FVM solutions to the system. The learning process was performed for the CFD data where the wall heat fluxes were 75, 85, 105, and 115 w/m^2^. The sensitivity tests were done for different accept ratio (AR) values (i.e. 0.3, 0.4, 0.5, 0.6, and 0.7) and inertia weight damping ratio (IWDR) values (i.e. 0.6, 0.7, 0.8, 0.9, and 0.99). After the best intelligence achievement, the accuracy of the PSOFIS for the prediction of the lost data was tested. One of the challenges of the CFD modeling is the lost data for a specific boundary condition. In this study, the CFD data were supposed to be available for wall heat fluxes of 75, 85, 105, and 125 w/m^2^, while there is no data for the wall heat flux of 95 w/m^2^. So, the PSOFIS learns the available CFD data and predicts the velocity profile for where the data is not available (i.e., wall heat flux of 95 w/m^2^). The CFD calculation was repeated for the wall heat flux of 95 w/m^2^ and the PSOFIS predictions compared with the CFD results.

The results of this study can be summarized as follows.

- The highest coefficient of determination and, as a result, the best intelligence is obtained for the AR and IWDR of 0.7 and 0.99, respectively.
- For the best intelligence, the regression number is about 0.98.
- The velocity profile of the PSOFIS corresponding to the learned data is the same as that of the CFD prediction.
- The PSOFIS can predict the velocity profile of the data, which is absent in learning, with the highest compatibility to the CFD prediction.
- For the performance assessment of the PSOFIS and for such a model, the learning (i.e., training and testing) time was 268 s, while the prediction of the CFD data lost was negligible (~ 1 s). In contrast, the CFD calculation takes around 600 s for each simulation.
