# Editorial Expression of Concern: Numerical investigation of nanofluid flow using CFD and fuzzy-based particle swarm optimization

**DOI:** 10.1038/s41598-022-16513-8

**Published:** 2022-07-14

**Authors:** Rahmad Syah, Marischa Elveny, Mahyuddin K. M. Nasution, Vadim V. Ponkratov, Mariya Yurievna Kuznetsova, Andrey Leonidovich Poltarykhin, Meisam Babanezhad

**Affiliations:** 1 Data Science & Computational Intelligence Research Group, Universitas Medan Area, Medan, Indonesia; 2 Data Science & Computational Intelligence Research Group, Universitas Sumatera Utara, Medan, Indonesia; 3 Financial University Under the Government of the Russian Federation, Moscow, Russian Federation; 4 Sechenov First Moscow State Medical University, Moscow, Russia; 5 Plekhanov Russian University of Economics, 117997 Moscow, Russia; 6 Department of Energy, Faculty of Mechanical Engineering, South Tehran Branch, Islamic Azad University, Tehran, Iran; 7 Department of Artificial Intelligence, Shunderman Industrial Strategy Co., Tehran, Iran

Editorial Expression of Concern to: *Scientific Reports* 10.1038/s41598-021-00279-6, published online 25 October 2021

The Editors are issuing an editorial expression of concern to alert readers that this article shows indication of irregularities in authorship. Concerns have been raised about the expertise of some of the authors not matching the subject matter of this article. Authors provided explanations regarding addition of authors during submission process, but they did not sufficiently document contributions of all authors.

Additionally, the code used to generate the results had not been previously made available. It is now deposited in the zenodo repository and can be found at: https://zenodo.org/record/6791248

Rahmad Syah, Marischa Elveny, Mahyuddin K. M. Nasution, Vadim V. Ponkratov and Meisam Babanezhad disagree with this Editorial Expression of Concern. Mariya Yurievna Kuznetsova and Andrey Leonidovich Poltarykhin did not respond to the correspondence from the Editors.

